# The Efficacy of Punch Biopsy and Diode Laser Combination in the Treatment of Pilonidal Sinus: A Comparative Study Across Different Patient Groups

**DOI:** 10.3390/jcm14093052

**Published:** 2025-04-28

**Authors:** Suat Evirgen, Sirin Cetin

**Affiliations:** 1 Department of General Surgery, Medical Faculty, Amasya University, Amasya 05100, Turkey; opdrse@gmail.com; 2 Department of Bioistatistical, Medical Faculty, Amasya University, Amasya 05100, Turkey

**Keywords:** pilonidal sinus, 1470 nm diode laser, punch biopsy

## Abstract

**Background:** This study aims to evaluate the efficacy and safety of a minimally invasive technique (pit excision with a punch biopsy needle) using a 1470 nm diode laser in the treatment of pilonidal sinus disease. **Methods:** A prospective study conducted included 187 patients who underwent laser treatment for pilonidal sinus. Patients were divided into two groups based on the severity of the disease: Group 1 (simple group) included patients who had no prior abscess formation and had one or two sinus openings, while Group 2 (complicated group) comprised patients with a history of abscess drainage and more two extensive sinus openings. The surgical procedure was performed under local anesthesia. The sinus openings were excised using a punch biopsy needle and subsequently closed with a diode laser probe. Patients were followed up at regular intervals during the postoperative period. **Results:** The findings revealed that the operative time (12.22 ± 1.72 min), postoperative pain scores and wound-healing duration were significantly lower in the simple group compared to the complicated group (*p* < 0.001). The recurrence rates were 3.7% in the simple group and 14.1% in the complicated group. The return-to-work time was 1.06 ± 0.32 days in the simple group and 1.32 ± 0.90 days in the complicated group. **Conclusions:** In conclusion, the combination of punch biopsy and diode laser is considered a safe and effective method, particularly for simple pilonidal sinus cases, due to its low complication rates, short recovery time, and early return-to-work advantages. However, more advanced treatment approaches are required for complicated cases.

## 1. Introduction

Pilonidal sinus disease is a chronic inflammatory condition that typically arises within the natal cleft of the sacrococcygeal region. Its estimated incidence is approximately 26 cases per 100,000 individuals [1]. The condition predominantly affects young adult males, particularly those between the ages of 15 and 30 years, and male sex is recognized as one of the primary risk factors for its development [2,3]. In addition, several other predisposing factors have been implicated in the pathogenesis of the disease, including obesity, prolonged sitting, excessive perspiration, dense body hair, and poor personal hygiene [4,5]. These factors are thought to contribute to follicular occlusion and localized inflammation, ultimately resulting in sinus tract formation.

An ideal treatment strategy for pilonidal sinus disease should be both technically simple and clinically effective. The selected approach is expected to offer several key advantages, including a short hospital stay, minimal tissue loss, low postoperative pain, reduced need for wound care, low recurrence rates, and favorable cosmetic outcomes [1,2]. Although numerous surgical and minimally invasive techniques have been described in the literature, none have succeeded in fully eliminating the risk of recurrence. Therefore, the choice of treatment should be tailored to the individual, taking into account the patient’s clinical presentation, lifestyle factors, and personal expectations, in order to optimize long-term outcomes and patient satisfaction.

The application of minimally invasive techniques in the management of pilonidal sinus disease dates back to the 1960s [3]. These approaches gained increasing attention in surgical practice following the introduction of the Bascom technique in the late 1980s [4] and have since been widely adopted in clinical settings. Although traditional excisional procedures aim to achieve early and complete recovery, even these methods do not guarantee a 100% success rate. Reported recurrence rates range between 0.4% and 1.6% for the Limberg flap and between 0.2% and 0.6% for the Karydakis procedure at 1- and 2-year follow-up intervals [5]. Despite these relatively low short-term rates, long-term outcomes—particularly at 5-year follow-up—show recurrence rates between 10% and 30%, and the risk of postoperative complications remains a significant concern. In response to these limitations, minimally invasive methods have been developed as alternative strategies, offering several potential advantages. For instance, in a large cohort study involving 1358 patients, Gips et al. [6] reported a 16.2% recurrence rate over a 7-year follow-up period following treatment with microsinusectomy, underscoring both the promise and the need for further refinement of these less invasive approaches.

Among the various minimally invasive techniques, endoscopic pilonidal sinus treatment (EPSiT), developed by Meneiro et al. and introduced to the literature through a multicenter study, has garnered considerable attention in recent years [7,8]. This technique involves endoscopic access to the pilonidal sinus cavity via a specially designed fistuloscope, performed under spinal anesthesia. During the procedure, necrotic tissue, granulation tissue, and embedded hair within the sinus are removed under direct visual guidance. One of the key advantages of EPSiT is the ability to perform thorough debridement under continuous endoscopic visualization, thereby enhancing procedural precision. Following debridement, the external sinus opening is widened to facilitate effective drainage and promote secondary intention healing. In the original study by Meneiro et al. [7,8], a complete wound-healing rate of 94.8% was reported among 44 patients, with an average healing time of 26.7 ± 10.4 days and a recurrence rate of only 5%. These findings support EPSiT as a safe, effective, and well-tolerated therapeutic option, particularly in patients seeking rapid recovery and improved postoperative comfort.

Despite the availability of numerous surgical techniques for the management of pilonidal sinus disease, a universally accepted gold-standard treatment has yet to be established [6]. An ideal surgical approach is expected to offer several key advantages, including minimal tissue excision, low recurrence rates, short hospitalization, early return to daily activities, minimal scarring, and ease of application [7]. In recent years, the growing adoption of minimally invasive techniques in surgical practice has brought particular attention to endoscopic procedures and diode laser-based therapies as promising alternatives for treating pilonidal sinus disease [8,9]. These modalities are associated with enhanced patient comfort, faster wound healing, and improved postoperative recovery, making them increasingly attractive options in clinical settings.

The primary objective of this study was to assess the efficacy and safety of a novel minimally invasive technique combining pit excision using a punch biopsy needle with diode laser ablation for the treatment of pilonidal sinus disease, based on a prospective patient series. Clinical outcomes were evaluated with a focus on postoperative complication rates and recurrence frequency. To further investigate the applicability and consistency of this approach across varying disease severities, patients were stratified into two groups: Group 1, consisting of early-stage, newly diagnosed cases, and Group 2, comprising complicated or late-stage presentations. This classification enabled a comparative analysis of treatment effectiveness and facilitated the evaluation of the method’s standardization potential for different clinical stages. Through this approach, the study aimed to determine the feasibility of implementing the technique across a spectrum of disease complexity.

## 2. Materials and Methods

### 2.1. Study Design and Patient Selection

A prospective observational study was conducted including 187 patients who un-derwent laser surgery for pilonidal sinus. Ethical approval was obtained from the Amasya Sabuncuoğlu Şerefeddin Training and Research Hospital Ethics Committee (Approval number: E-62949364-929-2022, 18 March 2022, and updated approval number: E-30640013-050.04-237646, 14 January 2025). The study was conducted in accordance with the principles of the Helsinki Declaration.

#### 2.1.1. Voluntary Participation and Confidentiality

All participants were given enough time to decide whether or not to participate in the study. They were told that they participated in the research voluntarily without any pressure or coercion and that they could leave the research at any time without giving any reason. All volunteers were informed about the conditions of confidentiality and participation in accordance with the relevant laws, and patients who gave their consent to participate were included in the study.

A total of 187 patients who underwent laser treatment for pilonidal sinus were included in the study. Patients with systemic colon diseases, such as inflammatory bowel disease, were excluded.

Patients were categorized into two groups based on clinical severity and prior medical history. Group 1 (Simple cases) included patients with no history of abscess formation, one or two closely located sinus openings, and sinus tracts confined to the midline (intergluteal sulcus). Group 2 (Complicated cases) consisted of patients with a history of abscess drainage, three or more sinus openings, or sinus openings extending 4 cm or more laterally from the midline. These classification criteria were established based on the existing literature to ensure clinical validity and reproducibility in distinguishing between simple and complicated cases.

Patients were divided into two groups based on the severity and onset of the disease:

Group 1: Patients with no prior abscess formation and one or two closely located sinus openings.

Group 2: Patients with a history of abscess drainage, more than three sinus openings, or sinus openings located more than 4 cm lateral to the intergluteal sulcus.

(Inclusion criteria were as follows: age ≥ 18 years, a primary diagnosis of pilonidal sinus disease, treatment with diode laser combined with punch biopsy, and provision of informed consent for a 6-month follow-up. Patients were excluded if they had systemic inflammatory or infectious diseases (e.g., Crohn’s disease, ulcerative colitis), a history of prior surgery in the same anatomical region, immunodeficiency disorders, or demonstrated non-compliance with follow-up visits).

#### 2.1.2. Inclusion and Exclusion Criteria

**Figure 1:**
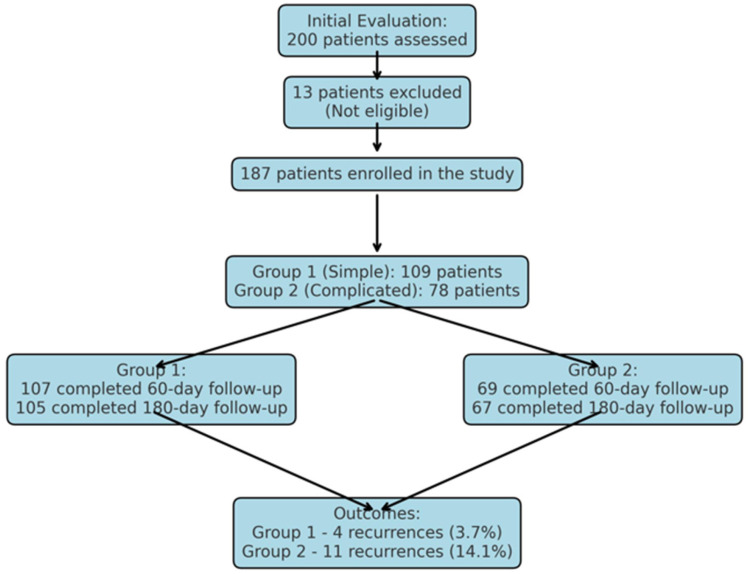
Patient flow and recurrence rates.

Patients aged 18 years or older with a primary diagnosis of pilonidal sinus disease who underwent minimally invasive surgical treatment consisting of pit excision using a 3–4 mm punch biopsy needle followed by diode laser ablation (1470 nm, 10 W) were eligible for inclusion. Only treatment-naïve individuals with no prior surgical interventions in the sacrococcygeal region were considered. Written informed consent was obtained from all participants, including agreement to adhere to the standardized 6-month postoperative follow-up schedule (days 1, 7, 15, 30, 45, 60, and 180). Patients were excluded if they had any systemic inflammatory or infectious diseases (e.g., Crohn’s disease, ulcerative colitis), immunodeficiency disorders, or were receiving immunosuppressive therapy. Additional exclusion criteria included prior surgical procedures involving the natal cleft, active local or systemic infections at the time of enrollment, pregnancy or lactation, and documented non-compliance with follow-up visits. These criteria were established to ensure a homogenous study population and minimize potential confounding factors affecting wound healing and recurrence rates. All procedures were performed under local anesthesia on an outpatient basis. The surgical protocol involved the use of a 1470 nm diode laser at 10 watts of power, delivering energy in 3 s pulses totaling approximately 30 joules, applied via a radial-fiber laser probe. The technique included the mechanical cleansing of the sinus tract and excision of the sinus opening using a punch biopsy, followed by advancement of the radial fiber into the tract and laser coagulation of the epithelial lining. Wounds were left open and managed with standard dressings; no primary closure was performed. Postoperative follow-up assessments were conducted on days 1, 7, 15, 30, 45, 60, and 180. During each visit, wound-healing status, presence of infection, signs of abscess formation, and any evidence of recurrence were systematically evaluated. Healing was defined as the complete epithelialization of the sinus openings and cessation of discharge (Figure 1).

In our study, we did not perform a prospective power analysis. However, we considered our current sample size (*n* = 187) to be relatively large compared to similar prospective studies, and therefore sufficient to detect statistically significant differences in key outcomes. For instance, the observed difference in recurrence rates between the simple and complicated groups was approximately 10%, which we estimated could be detected with over 80% power given our patient numbers. Nevertheless, we recommend that future studies include formal sample size calculations.

### 2.2. Surgical Procedure

**Figure 2:**
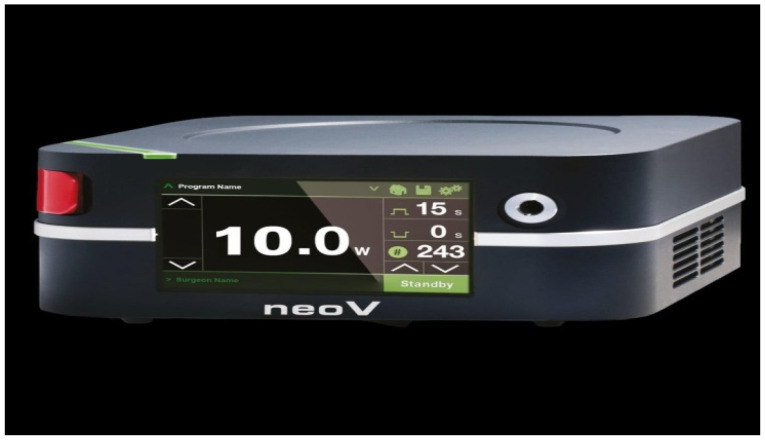
Diode laser device.

**Figure 3:**
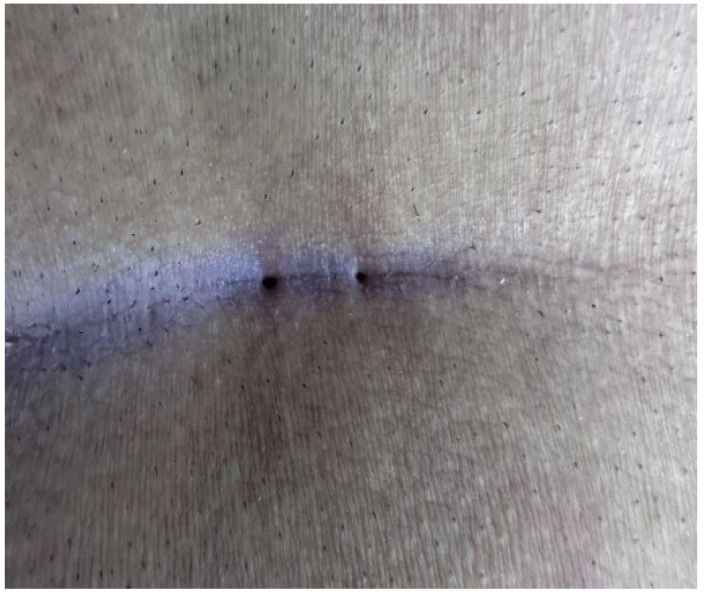
Two closely spaced sinus orifices (Group 1 patient).

**Figure 4:**
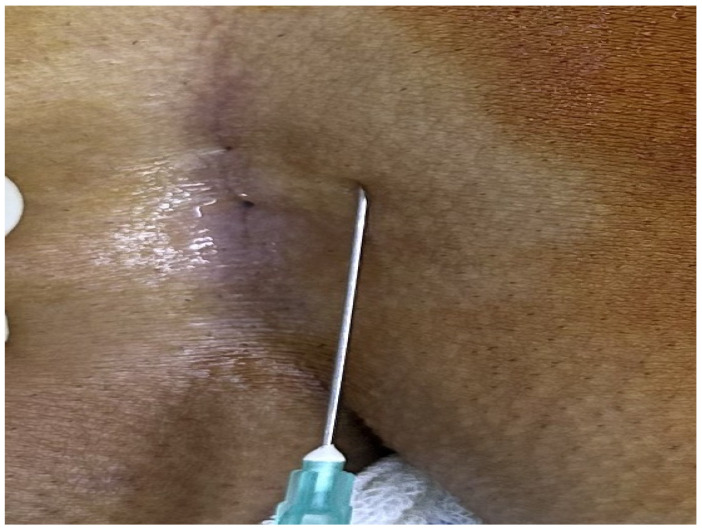
Application of local anesthesia.

**Figure 5:**
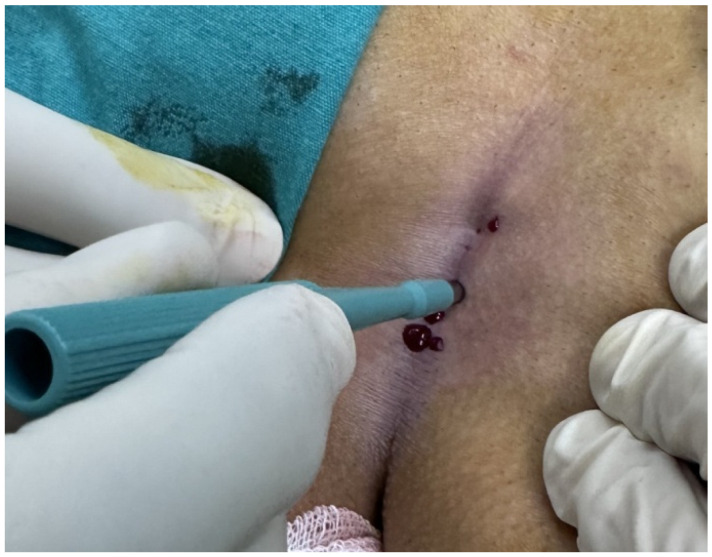
Excision of pit with punch biopsy needle.

**Figure 6:**
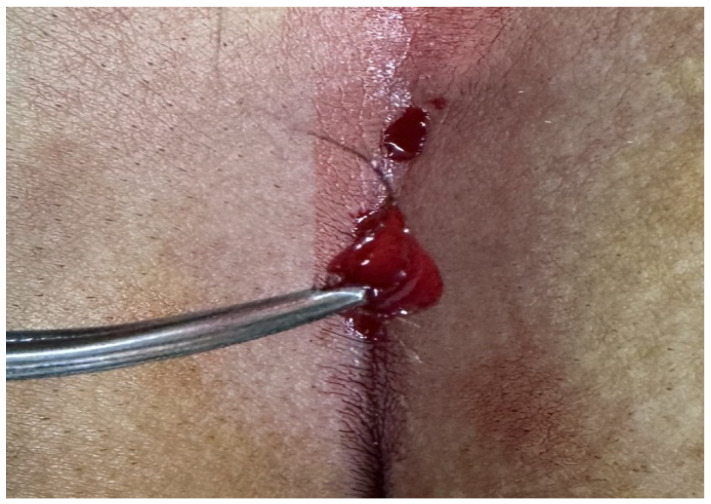
Hair removal.

**Figure 7:**
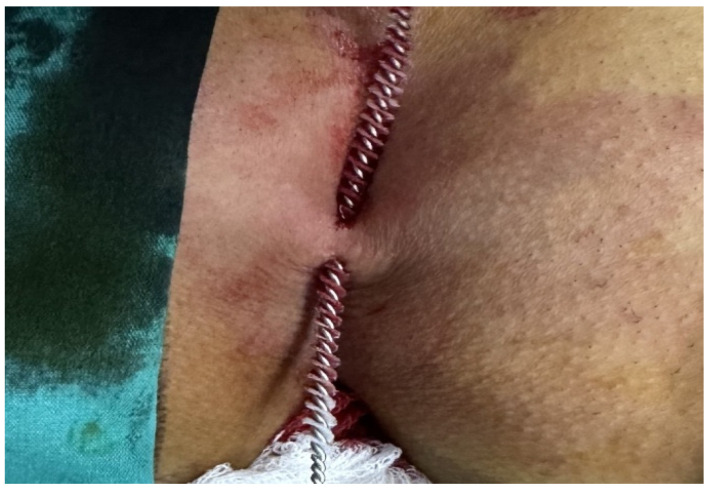
Cleaning of the sinus cavity using a special brush.

**Figure 8:**
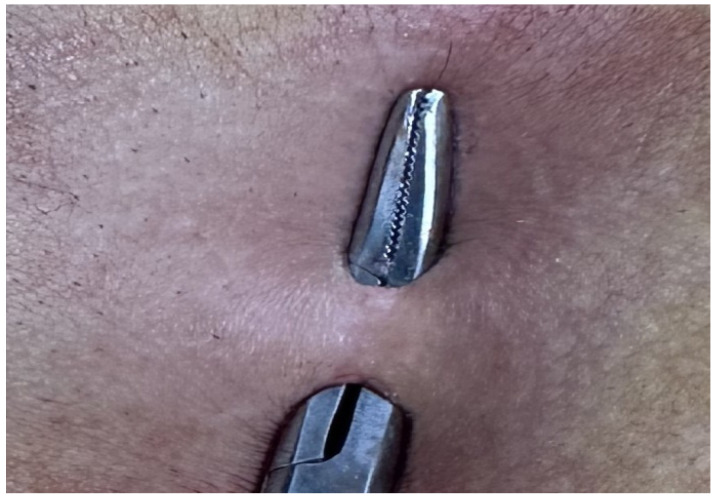
Relationship of the sinus orifices.

**Figure 9:**
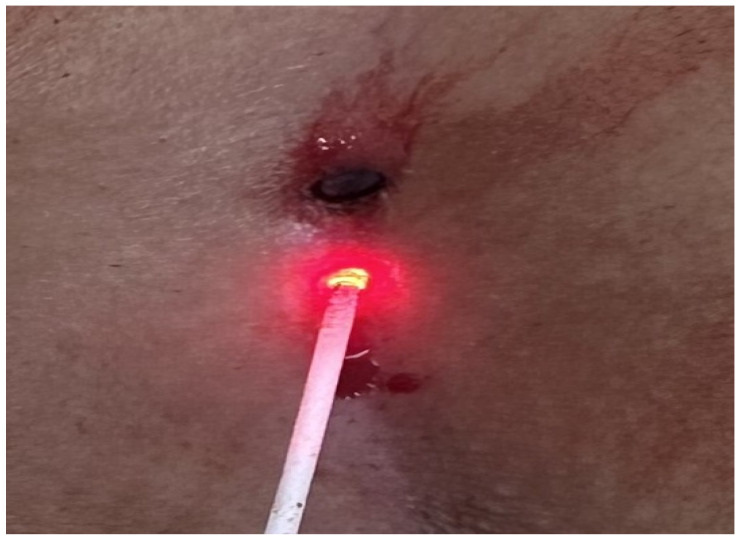
Application of diode laser.

**Figure 10:**
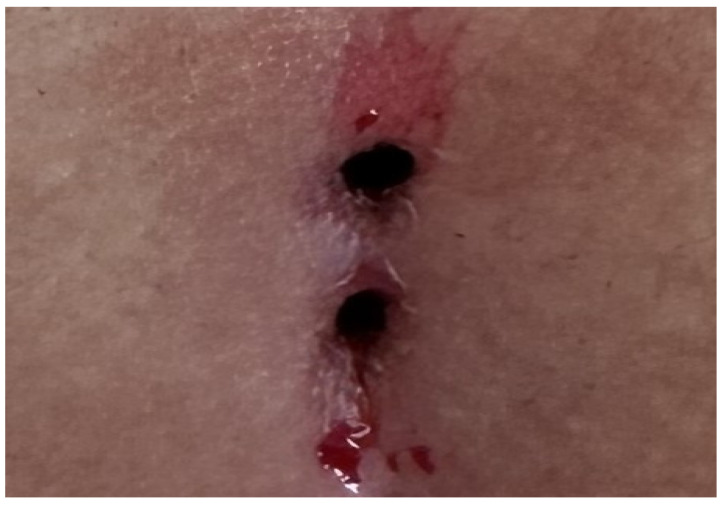
Appearance after laser application.

**Figure 11:**
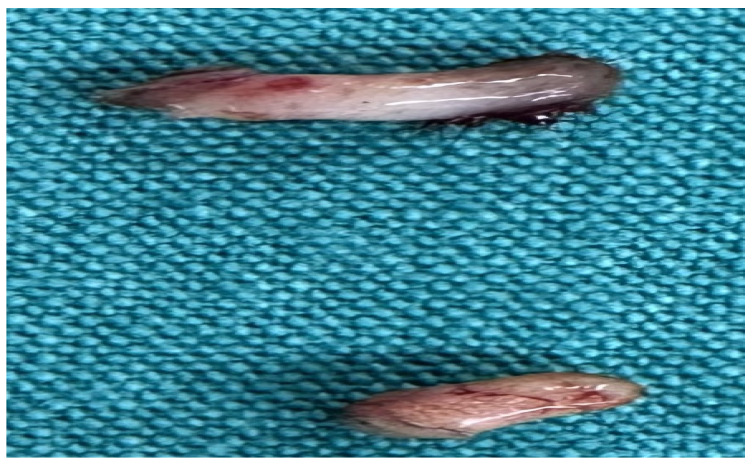
Pathology specimen.

The patients were operated on under local anesthesia in the operating room under appropriate field cleaning and sterile conditions, in the jackknife position. Prilocaine hydrochloride was used for local anesthesia. A single dose of cefazolin sodium was administered intravenously to the patients. The external sinus openings were excised using a 4 mm punch biopsy needle. After pit excision, the sinus cavity was cleaned with saline solution. The tissues within the sinus cavity were debrided using a specialized brush. Subsequently, the sinus cavities were uniformly closed using a diode laser probe (1470 nm, 10 watts, 3 s, 30 joules). The procedure was concluded by applying a dressing containing sodium fusidate ointment. Pain scoring was performed one hour postoperatively, and intravenous paracetamol was administered to all patients. The patients were discharged two hours later with a prescription for oral antibiotics (Figure 2, Figure 3, Figure 4, Figure 5, Figure 6, Figure 7, Figure 8, Figure 9, Figure 10 and Figure 11).

During the postoperative period, the patients were regularly followed up at intervals (on days 1, 7, 15, 30, 45, 60, and 180).

Day 1: Patients underwent their first follow-up, where early postoperative complications at the surgical site were assessed, dressings were changed, and detailed information was provided to the patients.

Day 7: Patients were called for a follow-up to evaluate potential complications such as infection, abscess, or bleeding at the surgical site.

Days 15, 30, and 45: Follow-ups were conducted to assess the stages of epithelialization.

Day 60: Non-healed wounds were identified as delayed healing during the 60-day follow-up.

Day 180: Patients were evaluated for recurrence during the 180-day follow-up.

### 2.3. Data Collection and Analysis

Operative time, postoperative pain, length of hospital stay, return-to-work time, wound-healing duration, and recurrence rates were evaluated. Pain scores were calculated using a numerical scale ranging from 0 to 10, and healing durations exceeding 60 days were considered as “delayed healing”.

### 2.4. Statistical Methods

The data obtained in this study were analyzed using SPSS 25 (Statistical Package for the Social Sciences) software. Continuous variables were expressed as mean ± standard deviation (mean ± SD), while categorical variables were expressed as numbers and percentages (%). Parametric and non-parametric tests were applied to evaluate differences between groups.

For the comparison of continuous variables between two groups, the Independent Samples t-test was used. The normality of the data distribution was assessed using the Kolmogorov–Smirnov and Shapiro–Wilk tests. Relationships between categorical variables were analyzed using the Chi-square test. Logistic regression analysis was applied to determine the relationship between postoperative complications and recurrence rates. The statistical significance level was set at *p* < 0.05.

To strengthen our statistical analysis, we not only reported *p*-values but also included effect sizes and confidence intervals to enhance the interpretability of our results. For instance, in our revised tables, we demonstrated that an operative time exceeding 12 min was associated with a nearly fourfold increase in recurrence risk (OR = 4.23), with a 95% confidence interval of 1.33–13.86. Similarly, we found that a wound-healing duration longer than 17 days increased the likelihood of recurrence by five times (OR = 5.06, 95% CI: 1.79–14.33).

In line with these methods, a statistical analysis of the study data was performed, and the results are presented in tables.

## 3. Results

The demographic characteristics of the patients are presented in Table 1. Recurrence rates were 3.7% in the simple group and 14.1% in the complicated group (*p* = 0.010). This discrepancy likely stems from the higher number of sinus orifices and prior surgical interventions in complicated cases, which may impair healing efficacy.

A statistically significant difference was observed between the simple and complicated groups in terms of recurrence development (*p* < 0.010; Table 2).

A statistically significant difference was identified between patients with and without recurrence in cases of postoperative abscess development (*p* < 0.015; Table 3).

Operative duration was significantly shorter in the simple group (8.4 ± 2.112.22 ± 1.72 min), whereas the complicated group exhibited markedly prolonged procedure times (13.2 ± 3.423.00 ± 2.79 min; *p* < 0.001; Table 4). This disparity may be attributed to the broader and deeper sinus tracts in complicated cases, necessitating more extensive surgical intervention.

Postoperative pain scores were significantly lower in the simple group (*p* = 0.037), supporting the efficacy of limited tissue damage and minimally invasive techniques. Wound-healing duration also differed substantially: the simple group demonstrated a mean healing time of 16.74 ± 2.54 days, compared to 27.99 ± 2.85 days in the complicated group (*p* < 0.001). These findings suggest that laser therapy facilitates accelerated recovery in early-stage disease. Recurrence rates were 3.7% in the simple group and 14.1% in the complicated group (*p* = 0.010). This discrepancy likely stems from the higher number of sinus orifices and prior surgical interventions in complicated cases, which may impair healing efficacy.

Return to normal daily activities occurred significantly faster in the simple group (1.06 ± 0.32 days) than in the complicated group (1.32 ± 0.90 days; *p* = 0.005), further underscoring the rapid recovery associated with laser therapy in early-stage patients.

When operative durations were analyzed, the simple group demonstrated a mean duration of 12.2 ± 1.72 12.22 ± 1.72 min, whereas the complicated group exhibited a significantly prolonged duration of 23.00 ± 2.79 23.00 ± 2.79 min. A statistically significant intergroup difference was identified (*p* < 0.001; Table 2).

Analysis of gender distribution revealed that the simple group consisted of 100 males (91%) and 9 females (9%), while the complicated group comprised 77 males (98%) and 1 female (2%). A statistically significant intergroup disparity was identified (*p* < 0.003; Table 2).

In the complicated group, 78 patients were initially enrolled; however, 69 of them attended the follow-up at day 60, and 67 completed the final follow-up at day 180. Similarly, in the simple group, out of 109 patients, 107 completed the 60-day evaluation, and 105 reached the 180-day follow-up. The loss of participants in both groups was attributed to patients who failed to complete their scheduled follow-up visits.

Postoperative pain in Table 2 refers to the number of patients who experienced significant postoperative pain, meaning pain that required additional analgesic intervention beyond the standard protocol.

**Figure 12:**
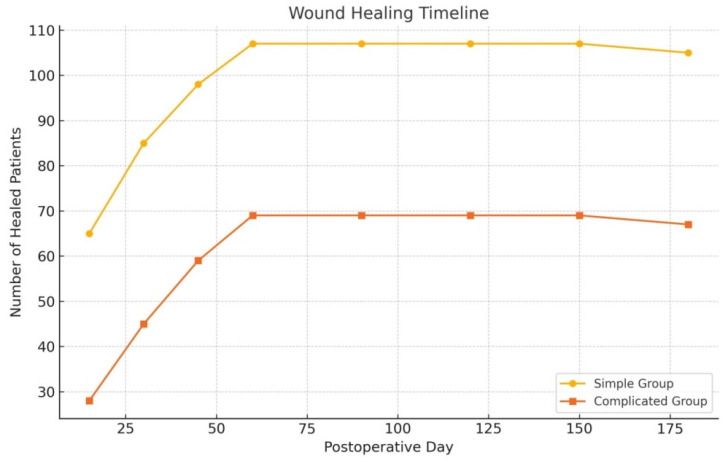
We considered wounds not healed by day 60 as “delayed healing” cases.

Patients with an operative time exceeding 12 min have a fourfold higher risk of recurrence. Those with wound-healing durations longer than 17 days have a fivefold higher risk of recurrence. Additionally, patients who develop postoperative abscesses have a 1.4-fold higher risk of recurrence (Table 4) (Figure 12).

## 4. Discussion

Collectively, these results indicate that diode laser therapy is a safe and effective therapeutic modality for simple pilonidal sinus cases, offering low complication rates, expedited return to daily activities, and enhanced patient comfort. However, the higher recurrence rates in complicated cases underscore the need to evaluate alternative surgical approaches or adjuvant therapies in this patient subgroup.

This study evaluated the efficacy and reliability of diode laser-assisted minimally invasive surgery in the treatment of pilonidal sinus disease. The findings demonstrated that the laser technique offers advantages such as shorter hospital stays, lower postoperative pain scores, and earlier return to daily activities. Specifically, the combination of punch biopsy for pit excision with laser ablation ensured homogeneous closure of sinus tracts and accelerated wound healing. Prior studies have associated laser methods with lower complication and recurrence rates compared to open surgical techniques [8,9]. However, the observed disparity in complication rates between simple and complicated groups in this study underscores the influence of disease severity on treatment outcomes.

While this approach capitalizes on the benefits of minimally invasive surgery, it also highlights the need for standardization. Future large-scale studies comparing diverse laser devices, energy settings, and surgical techniques are warranted. Additionally, patient education and optimized postoperative care are critical factors that may further enhance therapeutic success.

Operative duration was significantly shorter in the simple group (12.3 vs. 23 min, *p* < 0.001), aligning with Yardımcı et al. [10], though their study lacked subgroup stratification. Postoperative pain was mild in 96.3% of simple and 88% of complicated cases, consistent with Georgiou [11]. All patients were discharged within 2 h (except one with bleeding), contrasting with Hassan et al. [12], who reported 4–6 h stays.

Wound infection rates were significantly lower in the simple group (1.8% vs. 11.5%, *p* < 0.001), compared to 8% in De Decker et al. [13]. Return-to-activity time averaged 1.06 days (simple) vs. 1.32 days (complicated), shorter than Yardımcı et al. [10] (2.6 days). Recurrence rates (3.7% vs. 14.1%, *p* = 0.010) were lower than Bilgin et al. [14] (11%), reflecting the benefits of subgroup stratification

Interest in laser applications for pilonidal sinus disease has grown markedly over the past decade. While Nd-YAG lasers were historically utilized for hair removal in such patients, recent advancements include the use of Alexandrite (755 nm) and Nd-YAG (1064 nm) lasers for sinus tract ablation, albeit with recurrence rates of 13–19% and requiring multiple sessions [9,15,16]. Dessily et al. [1] and Pappas et al. [17] advocated for the 1470 nm diode laser, reporting low morbidity and recurrence rates (237 cases).

In this study, patients were stratified into two groups: simple group: no abscess history, ≤2 closely spaced sinus orifices, no prior surgery; complicated group: abscess drainage, recurrence, ≥3 sinus orifices, lateral sinus tracts > 4 cm from the intergluteal sulcus, or inter-orifice distance > 5 cm.

All patients received preoperative intravenous cefazolin sodium and underwent surgery under local anesthesia in an operating room. Sinus orifices were excised using a 3–4 mm punch biopsy needle, followed by 1470 nm diode laser ablation (10 W, 3 sec pulses, 30 J). Sinus tracts were debrided with a specialized brush, irrigated with saline, and closed via retrograde laser-probe withdrawal. Postoperative care included ice application, fusidic acid ointment, intravenous paracetamol, and discharge with oral cefixime. Outcomes assessed included operative duration, healing rates, recurrence, wound infections, abscess formation, hospital stay, return-to-activity time, and pain scores. Follow-up evaluations occurred at 1, 7, 15, 30, 45, 60, and 180 days.

We compared our 180-day recurrence rates with those reported for endoscopic pilonidal sinus treatment (EPSiT) and other laser-based modalities. In our study, the diode laser combined with punch biopsy yielded a recurrence rate of 3.7% in the simple group and 14.1% in the complicated group after six months of follow-up. In the literature, a multicenter study by Meinero et al. [7,8] on the EPSiT technique reported a recurrence rate of approximately 5% after a one-year follow-up, with complete wound healing achieved in 94–95% of patients and an average healing time of around four weeks. Additionally, in a large series by Pappas and Christodoulou investigating the laser-based Sinus Laser Ablation Treatment (SiLaT), successful healing was reported in 90.3% of 237 patients after the first laser session, corresponding to a recurrence rate of 9.7%. Notably, their cohort included patients with complicated disease characteristics, and recurrence rates remained below 10% even at one year. In comparison, while our recurrence rate of 14.1% in the complicated group appears slightly higher than that of EPSiT, it remains within the acceptable range reported for laser-based interventions. It is important to emphasize that complicated cases inherently carry a higher risk of recurrence due to the presence of multiple sinus tracts and a history of abscess formation, regardless of the treatment modality. Indeed, the literature indicates that even conventional surgical techniques may yield recurrence rates of up to 20% in similar patient populations.

Although the initial acquisition cost of diode laser devices is relatively high, the procedure’s practical advantages, such as being performed under local anesthesia, having a short operative duration, requiring no hospitalization, and allowing patients to return quickly to daily activities, offer significant benefits. Particularly when compared with conventional excision and primary closure techniques, which are often associated with longer work absences and more intensive wound care, laser treatment may provide indirect economic advantages. As our study was focused on clinical outcomes, we did not conduct a formal cost analysis. However, we believe that future research should assess the long-term cost effectiveness of laser therapy from both healthcare system and patient perspectives. Evaluating the acquisition, maintenance, and training costs associated with laser equipment across different healthcare settings and comparing them with traditional surgical methods would offer valuable guidance for policy makers and clinical decision makers.

Using EPSiT (endoscopic pilonidal sinus treatment), Meinero et al. had ~5% recurrence in their multicenter series [8], and with laser ablation techniques like the SiLaT procedure, Pappas et al. reported roughly 9–10% recurrence in a large cohort [17]. In a study conducted by Dessily et al. in 2017, the success rate of pilonidal sinus destruction using a radial laser probe was reported to be 87.5% [1]. 

The International Pilonidal Disease Association has introduced a comprehensive four-stage staging system that integrates clinical features, symptomati c profiles, therapeutic interventions, and recurrence patterns. This framework enhances the stratification of disease severity and aligns more precisely with evidence-based clinical management strategies [18].

Prospective studies conducted across multiple centers and by different surgeons would be instrumental in evaluating both the reproducibility and learnability of our technique. In particular, a randomized controlled trial design comparing our minimally invasive method with conventional surgical techniques (such as excision with primary closure or flap procedures) could most robustly elucidate the advantages and limitations of this approach. However, our study has certain limitations. First, the single-center nature of the study and the fact that all procedures were performed by a single surgeon may limit the generalizability of the findings. While this allowed for consistency in technique and minimized the impact of the learning curve, it remains to be tested whether similar outcomes can be achieved by different practitioners. Second, the non-randomized design and absence of a control group introduce a risk of selection bias; for instance, patient treatment preferences or referral patterns to our clinic may have influenced the results. We have acknowledged this potential bias in the interpretation of our findings. Third, our follow-up period was relatively short (6 months), which may be insufficient for capturing long-term recurrence rates, and future studies with extended follow-up durations are warranted.

## 5. Conclusions

In patients with newly diagnosed pilonidal sinus, particularly those without prior abscess formation and presenting with one or two closely located sinus openings, the use of 1470 nm diode laser therapy following pit excision with a punch biopsy needle has emerged as a highly effective and well-tolerated treatment modality. This minimally invasive technique offers several practical advantages, including ease of application, limited tissue trauma, and a low complication rate, owing to the simplicity of the surgical procedure. The data from our study demonstrated that this method resulted in reduced postoperative pain, early return to work, and low recurrence rates. Additionally, rapid resumption of daily activities and favorable cosmetic outcomes further contributed to high levels of patient satisfaction. Given these positive clinical outcomes, laser-assisted minimally invasive surgery may be considered a safe and effective first-line treatment option for patients with early-stage pilonidal sinus disease. This approach not only improves individual patient care but may also contribute to reduced healthcare utilization by minimizing the need for prolonged follow-up and hospital-based interventions.

### Limitations in Complex Cases

Current evidence suggests that laser-assisted techniques demonstrate certain disadvantages compared to simple cases when managing complicated pilonidal sinus presentations. These include patients with multiple sinus tracts, recurrent infection history, or extensive deep-seated lesions. Our findings indicate significantly higher recurrence rates and increased postoperative complication risks in this patient population. These observations suggest that diode laser therapy may yield less favorable outcomes in cases with complex anatomical features or advanced disease stages compared to uncomplicated presentations. Future Directions: the following technical modifications warrant further investigation for this challenging subgroup: the optimization of energy parameters and prolonged application durations.

With hybrid approaches combining laser with conventional excisional methods, large-scale multicenter comparative studies are imperative to establish optimal treatment protocols for complex cases. Such research initiatives may enhance clinical success rates while mitigating recurrence and complication risks.

## Figures and Tables

**Table 1:** Demographic features of patients.

Characteristic	Simple Group (*n* = 109)	Complex Group (*n* = 78)
Age (mean ± SD)	25.6 ± 4.1	26.4 ± 3.8
Gender (M/F)	81/28	59/19
Body Mass Index	23.7 ± 2.5	24.1 ± 2.9
Smoking (%)	15 (13.8%)	23 (29.5%)
Number of Sinuses	1.7 ± 0.6	3.2 ± 0.9

**Table 2:** Differences between simple and complicated groups.

	Simple (*n* = 109)	Complicate (*n* = 78)	*p*
Operative Time	12.22 ±1.72	23.00 ± 2.79	<0.001
Postoperative Pain	4/105	9/69	0.037
Hospital Stay Duration	2 h	2 h	-
Return-to-Work Time	1.06 ± 0.32	1.32 ± 0.90	0.005
Wound Healing	16.74 ± 2.54	27.99 ± 2.85	<0.001
Postoperative Abscess Development	2/107	9/69	0.007
Recurrence	4/105 (Number of patients on day 180)	11/67 (Number of patients on day 180)	0.010

**Table 3:** The recurrence rates between the simple and complicated groups.

	Recurrence Present	Recurrence Absent	*p*
Postoperative Abscess Present	3	8	0.015
Postoperative Abscess Absent	12	164	

**Table 4:** Multivariate logistic regression model determining the association between simple and complicated groups.

Variable	Odds Ratio	95% CI	*p*-Value
Operation Time > 12 min	4.23	1.33–13.86	0.005
Wound Healing Time > 17 days	5.06	1.79–14.33	0.001
Postop Abscess Formation	1.40	1.45–3.91	0.009

## Data Availability

The data supporting the results of this study are available from the corresponding author upon reasonable request via email. However, their use is subject to compliance with ethical guidelines and may require approval from the institutional review board.
